# Dried fruit intake and lower risk of type 2 diabetes: a two-sample mendelian randomization study

**DOI:** 10.1186/s12986-024-00813-z

**Published:** 2024-07-10

**Authors:** Jianbin Guan, Tao Liu, Kaitan Yang, Hao Chen

**Affiliations:** 1 Honghui Hospital, Xi’an Jiaotong University, Xi’an, Shannxi 710054 China; 2 Truma Rehabilitation Department, Honghui Hospital, Xi’an Jiaotong University, Xi’an, Shannxi 710054 China

**Keywords:** Type 2 diabetes, Dried fruit, Causal relationship, Incidence risk, Single-nucleotide polymorphisms, Mendelian randomization

## Abstract

**Background:**

Previous studies have shown controversy about whether dried fruit intake is associated with type 2 diabetes. This study aimed to examine the potential causal effect of dried fruit intake on type 2 diabetes by conducting a two-sample Mendelian randomization study.

**Methods:**

We used genome-wide association study (GWAS) summary statistics for MR analysis to explore the causal association of dried fruit intake with T2D. The inverse-variance weighted (IVW) method was used as the main analytical method for MR analysis. In addition, the MR-Egger method and the weighted median method were applied to supplement the IVW method. Furthermore, Cochrane’s Q test, MR-Egger intercept test, and leave-one-out analysis were used to perform sensitivity analysis. The funnel plot was used to assess publication bias.

**Results:**

The results from the IVW analysis indicated that dried fruit intake could reduce the risk of T2D [odds ratio (OR) = 0.392, 95% confidence interval (CI): 0.241–0.636, *p*-value = 0.0001]. In addition, the result of additional method Weighted median is parallel to the effects estimated by IVW. Furthermore, the sensitivity analysis illustrates that our MR analysis was unaffected by heterogeneity and horizontal pleiotropy. Finally, the results of the leave-one-out method showed the robustness of our MR results. And the funnel plot shows a symmetrical distribution.

**Conclusion:**

Our study provides evidence for the benefits of dried fruit intake on T2D. Therefore, a reasonable consumption of dried fruit may provide primary prevention.

**Supplementary Information:**

The online version contains supplementary material available at 10.1186/s12986-024-00813-z.

## Introduction

The consumption of dried fruits has been a topic of debate in relation to its potential impact on type 2 diabetes (T2D) [1]. Dried fruits, cherished for their concentrated flavors and prolonged shelf life, have emerged as a popular choice among individuals seeking healthier snack alternatives. However, concerns have been raised due to their high natural sugar content, particularly in the context of T2D—a metabolic disorder characterized by impaired insulin function and elevated blood sugar levels [2]. T2D, which constitutes the majority of diabetes cases globally, presents significant public health challenges due to its association with various complications, including cardiovascular disease, kidney dysfunction, and nerve damage [2]. Given the pivotal role of dietary choices in managing blood sugar levels [3], the inclusion of dried fruits in the diets of individuals with T2D has elicited both enthusiasm and caution. On one hand, dried fruits offer essential nutrients such as vitamins, minerals, and dietary fiber, potentially enriching a balanced diet. The fiber content in dried fruits holds promise for improving blood sugar control and enhancing digestive health [4]. However, the concentrated sugars in dried fruits, released more rapidly into the bloodstream compared to their fresh counterparts, may precipitate rapid spikes in postprandial glycemia, posing challenges for individuals striving to maintain stable glucose levels [5].

Observational studies focus solely on the correlation between exposure and outcome, lacking the ability to infer causal relationships. Additionally, both observable and unobservable residual confounders in observational studies may introduce bias or lead to opposite conclusions. To overcome these limitations, novel research methods such as Mendelian Randomization (MR) have been developed [6]. MR studies utilize single nucleotide polymorphisms (SNPs) as instrumental variables (IVs) to estimate causal associations between exposures and outcomes of interest [7]. SNPs conform to the principle of random assignment of genetic variants at meiosis, which avoids the effect of confounding factors and the potential impact of reverse causation since genetic variants precede the onset of disease [8]. Recent MR studies have revealed the impact of lifestyle behaviors on T2D risk and longevity, offering insights into potential influential exposure factors for T2D [9, 10]. Building on this foundation, our study employs a two-sample MR design to investigate the causal correlation between dried fruit intake and T2D, aiming to provide scientific evidence for the primary prevention of T2D.

## Materials and methods

### Study design

**Fig. 1:**
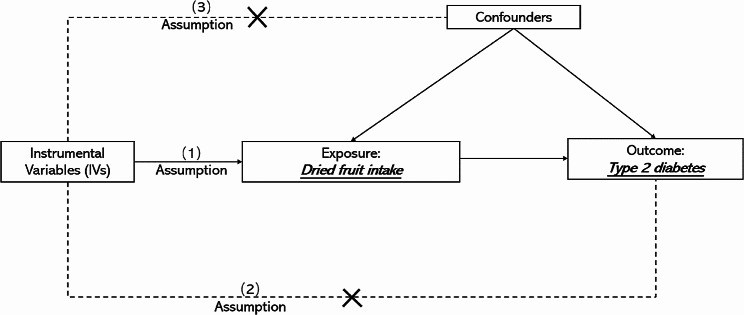
Flow diagram for Mendelian randomizationstudy

A flow diagram illustrating the entire study process is depicted in Fig. 1. To meet the assumptions required for MR studies, three main criteria were addressed: (1) Instrumental variables (IVs) must be strongly associated with exposure factors, (2) IVs must be independent of confounding factors, and (3) IVs must be solely associated with outcomes through exposure factors.

### Date sources

The Genome-Wide Association Study (GWAS) data for dried fruit intake were derived from a large cohort study involving approximately 500,000 individuals conducted by the UK Biobank. The study collected genotypic and various phenotypic data and was approved by the research ethics committee. Participants in the cohort were invited to the local evaluation center for data collection using a touch-screen questionnaire or standardized anthropometry. Participants’ intake of dried fruits as an exposure factor was extracted by a questionnaire asking about the frequency of dried fruit intake. The participants were asked, “How many pieces of dried fruit would you eat per day?” (One prune, one dried apricot and ten raisins are considered as one piece). In addition, three additional options, (1) less than one, (2) do not know, and (3) prefer not to answer, were available for participants to select. In total, 421,764 European participants’ dried fruit intake data were obtained. The GWAS summary statistics have been included in the IEU OpenGWAS database and are easily available for researchers to download (accession number: ukb-b-16,576) [11]. Genome-wide association study summary statistics for T2D were derived from the IEU GWAS of 61,714 cases and 593,952 controls [12].

### Instrumental variable selection

To identify SNPs significantly associated with dried fruit intake as valid IVs, we chose a cutoff *p*-value < 5 × 10^− 8^ as genome-wide significance. In addition, SNPs within a window size of 10,000 kb at a threshold of r^2^ < 0.001 were pruned to mitigate linkage disequilibrium (LD). In addition, we downloaded GWAS summary data of fasting glucose, fasting insulin and BMI, removing SNPs from IVs that were closely associated (*p*-value < 5 × 10^− 8^) with these confounders. Sources of the GWAS summary statistics for the confounders are shown in Supplementary Table 1. Finally, we calculated the F-statistic to assess the degree of weak instrumental bias (Supplementary Table 2). If the F statistic > 10, it was considered that no bias was caused by weak IVs [13].

### Statistical methods

The inverse-variance weighted (IVW) method was used as the main analytical method for estimating potential causal effects, which is an extension of the Wald ratio estimator based on the principles of Meta-analysis [14]. In addition, the MR-Egger method and the weighted median method were applied to supplement the IVW method [15, 16]. These three approaches are considered the most scientific and commonly used methods, providing robust analysis for MR studies. The criterion for using the weighted median method is that at least 50% of the SNPs must satisfy the premise that they are valid IVs [16]. The MR-Egger method provides unbiased estimates even when all selected IVs are multivariate [15]. Results of causal associations were presented as odds ratios (OR) and 95% confidence intervals (95% CI). Cochrane’s Q values were used to assess heterogeneity. MR-Egger intercept test was utilized to detect horizontal pleiotropy [17, 18]. In addition, the leave-one-out analysis was performed to assess the robustness of the results.

All statistical analyses were conducted using the “Two Sample MR” (version 0.5.7, Stephen Burgess, Chicago, IL, USA) and “Mendelian Randomization” (version 0.8.0) in the statistical program R (version 4.3.1). *p* < 0.05 was considered as statistically significant. The study protocol and details were not pre-registered.

## Results

### Instrumental variable details

A total of 43 SNPs were initially identified as independent and strongly associated with dried fruit intake. After excluding SNPs associated with confounding factors, 36 SNPs were retained as instrumental variables (IVs). Details of these 36 IVs are provided in Supplementary Table 2. The F-statistic for these IVs was 15.39, indicating their solid potential to predict dried fruit intake levels. Notably, all IVs exhibited stronger associations with dried fruit exposure than with T2D outcomes (Supplementary Table 3).

### Causal effects of dried fruit intake on T2D

**Fig. 2:**
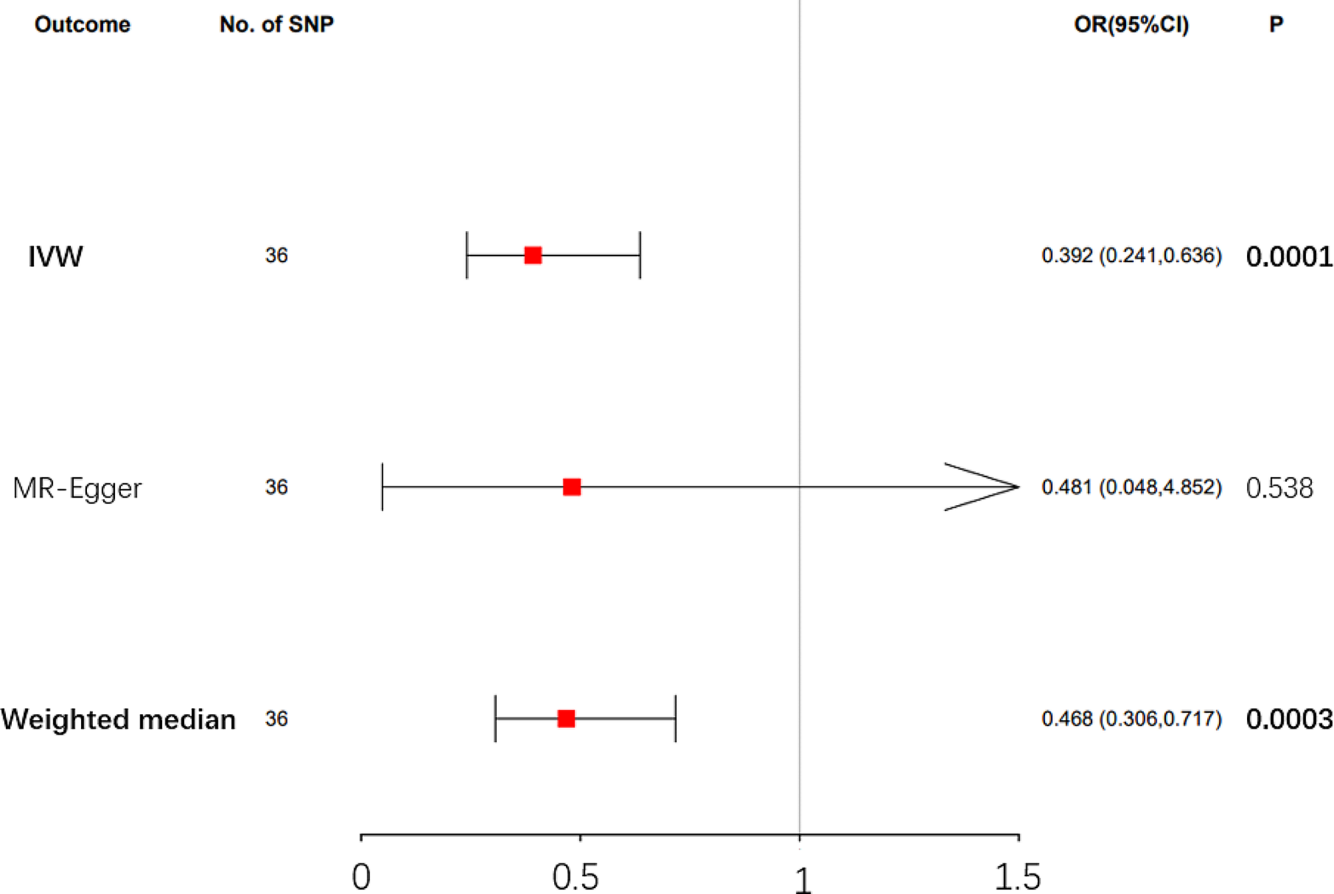
Causal effects of dried fruit intake on T2D assessed by the inverse-variance weighted (IVW) method, MR-Egger method, and Weighted median method

**Fig. 3:**
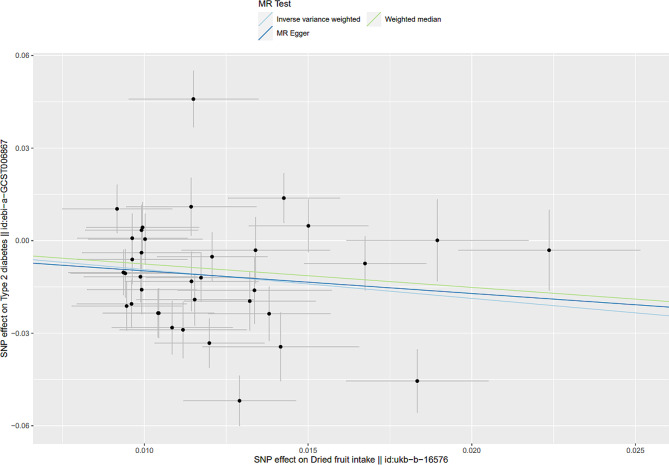
Scatter plot of genetic correlations of dried fruit intake and T2D using different MR methods

Mendelian randomization results from the IVW method suggested a causal association between dried fruit intake and T2D. Specifically, higher dried fruit intake was associated with a decreased risk of T2D. The risk of T2D decreased by 60.8% (OR = 0.392, 95% CI: 0.241–0.636, *p*-value = 0.0001, Fig. 2) for every increase of dried fruit intake by one standard deviation (1.275 pieces a day). Subsequent application of the MR Egger and weighted median methods corroborated these findings, indicating consistent estimates of the causal association between dried fruit intake and T2D risk (Table 1; Fig. 3).

**Table 1:** Causal effects of dried fruit intake on T2D evaluated by IVW method, MR Egger method, and weighted median method

Outcomes	Methods	Beta	SE	OR (95% CI)	*P*-value
T2D	IVW	-0.936	0.247	0.392 (0.241,0.636)	0.0001
	MR Egger	-0.732	1.179	0.481 (0.048,4.852)	0.538
	Weighted median	-0.758	0.217	0.468 (0.306,0.717)	0.0003

T2D, type 2 diabetes; SE, standard error; IVW, inverse variance weighted

### Sensitivity analysis

**Fig. 4:**
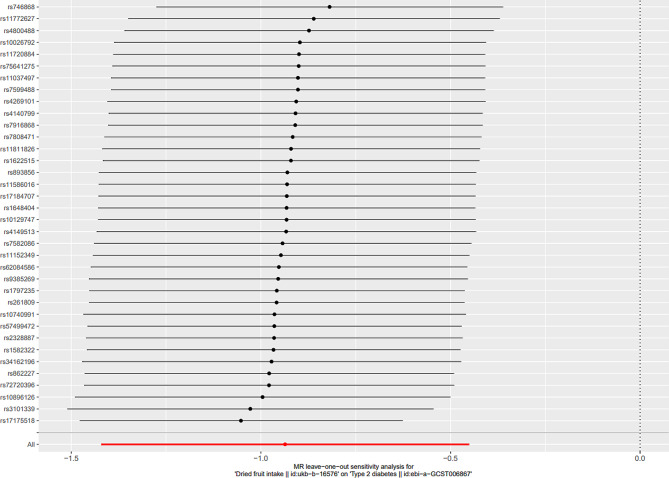
Leave-one-out analysis for dried fruit intake on T2D

**Fig. 5:**
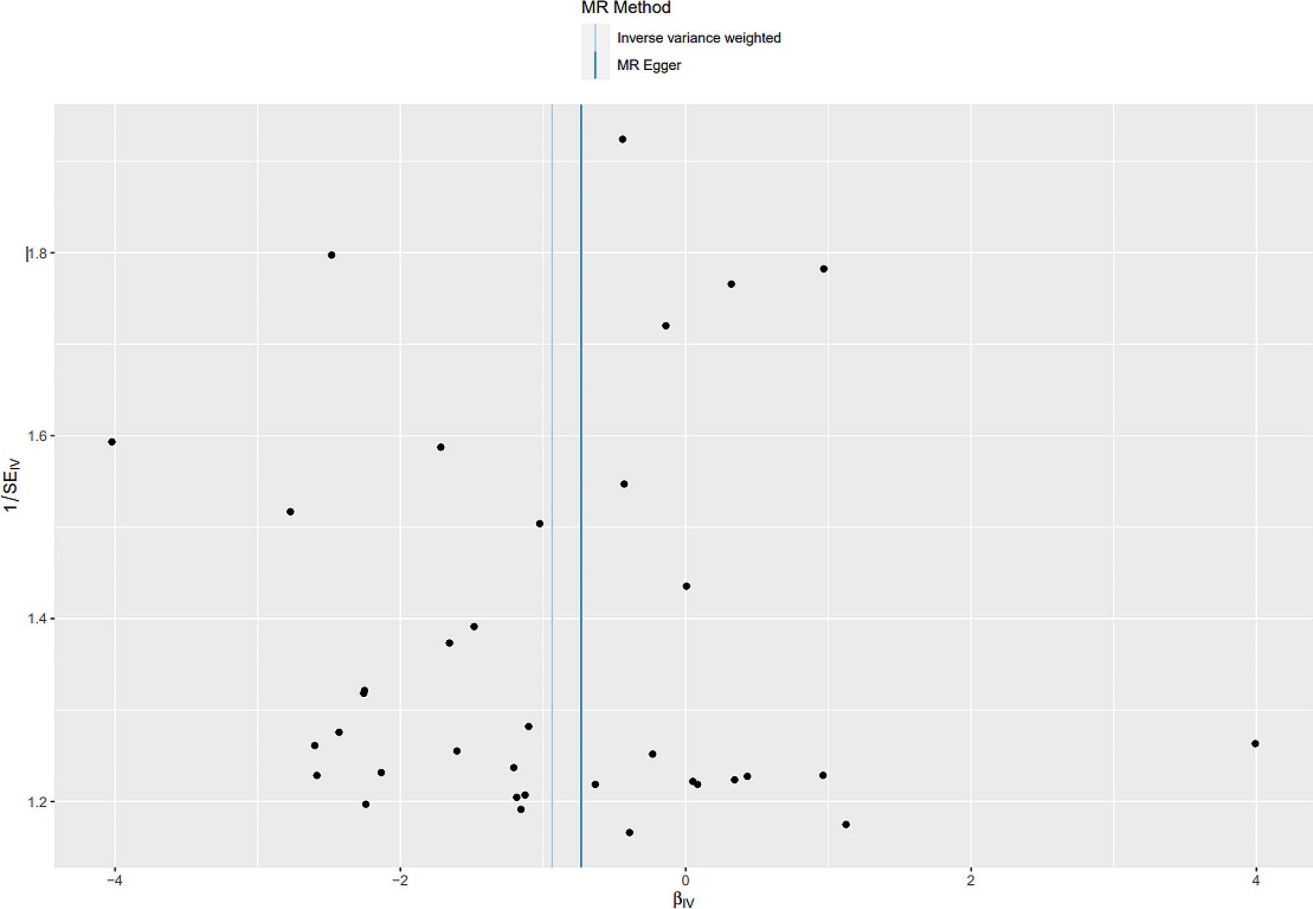
Funnel plot for dried fruit intake on T2D

Cochran’s Q-test revealed significant heterogeneity among the 36 IVs (Q_pval_IVW_ = 7.840342e^− 16^, Q_pval_MR−Egger_ = 3.904277e^− 16^) (Table 2). However, the MR-Egger intercept test suggested no evidence of horizontal pleiotropy between dried fruit intake and T2D (MR-Egger intercept = -0.0025, *p*-value = 0.86). Furthermore, leave-one-out analysis demonstrated the stability of the MR results, as excluding any one IV did not significantly alter the overall findings (Fig. 4). Additionally, the funnel plot exhibited a symmetrical distribution (Fig. 5).

**Table 2:** Results of heterogeneity by Cochran’s Q test

Outcome	Method	Cochran’s Q test		
		Q	Q_df	Q_pval
T2D	IVW	147.91	34	7.84e-^16^
	MR-Egger	147.77	35	3.904e-^16^

T2D, type 2 diabetes; IVW, inverse variance weighted

## Discussion

### Reappraisal of dried fruit consumption in relation to T2D risk

In light of historical medical perspectives, the consumption of dried fruit was previously discouraged due to concerns regarding their high fat and sugar content. However, recent years have witnessed a shift in this view. Conventional dried fruits are now recognized for their notable fiber content, minimal fat presence, and concentration of diverse micronutrients. Additionally, they offer enhanced convenience and extended shelf life compared to their fresh counterparts [19]. Evidence from numerous randomized clinical trials and animal studies underscores the potential advantageous impact of dried fruits in mitigating cardiovascular diseases [20, 21]. Dried fruits represent a rich source of macro and micronutrients, accompanied by significant bioactive compounds, which collectively possess the potential to influence and modulate distinct metabolic ailments [21–23]. Nonetheless, the linkage between dried fruit consumption and T2D has been scarcely explored in existing research. However, the linkage between dried fruit consumption and T2D has been scarcely explored in existing research, leaving the precise involvement of dried fruit in the onset and progression of T2D subject to dispute [24, 25]. Addressing this gap, our study represents a pioneering effort in the field of MR study, being the first MR investigation aimed at evaluating the causal impact of dried fruit consumption on the development of T2D. Our study rigorously adhered to the three main assumptions of MR analysis. Firstly, a strict significance threshold (*p*-value < 5 × 10^− 8^) was applied to screen SNPs associated with dried fruit intake as IVs. In addition, we eliminated the linkage disequilibrium of IVs. Additionally, we pruned SNPs in linkage disequilibrium and excluded those strongly associated with confounding factors. Secondly, we ensured that selected IVs were more strongly correlated with dried fruit exposure than with T2D outcomes. Finally, we utilized the MR-Egger intercept test to assess and mitigate the potential influence of horizontal pleiotropy, confirming the robustness of our results. Findings from our MR analysis suggest a causal association between dried fruit intake and a reduced risk of T2D.

### Potential mechanisms underlying the Protective effects of dried fruits

Several potential mechanisms may underlie this association. Data from the National Health and Nutrition Examination Survey indicate that dried fruit consumption is correlated with positive outcomes such as enhanced nutrient intake, improved diet quality scores, and reduced BMI, factors known to influence T2D risk [26–29]. Elevated blood sugar levels can impair the functionality of pancreatic β-cells, leading to their dysfunction and contributing to the development of insulin resistance, which is a hallmark of type 2 diabetes. Interestingly, research suggests that hawthorn fruit extract may offer protective effects against this process. Specifically, studies have shown that hawthorn fruit extract can alleviate oxidative stress and endoplasmic reticulum stress induced by high fructose levels, particularly within pancreatic β-cells [30]. Dried fruit is a matrix of important bioactive compounds such as Vitamins (Vitamin E, niacin, choline and/or folic acid) [31], micronutrient minerals (magnesium, potassium, calcium and/or phosphorus) [32], phenolic compounds, and carotenoid [33]. Certain constituents within dried fruits have the potential to exert a pivotal influence on mitigating the risk of T2D. For example, carotenoids present in dried fruits possess inherent antioxidant attributes that may contribute to diminishing the susceptibility to diabetes. Notably, augmented dietary intake of carotenoids is correlated with a lowered risk of developing T2D, underscoring the significant relationship between higher carotenoid consumption and risk reduction [34]. Dried fruits stand out due to their substantial β-carotene content, a factor that has been extensively linked to a protective role against the development of T2D [27, 35]. Furthermore, these fruits predominantly consist of carbohydrates, with relatively low proportions of protein and fat. Importantly, both dried fruits and their counterparts comprise a notable quantity of dietary fiber. This distinctive and diverse nutritional composition positions dried fruits as essential dietary components to mitigate the risk of various metabolic disorders [36].

### Therapeutic potential of flavonoids and grape polyphenols in T2D management

Flavonoids are a class of bioactive compounds found in various plant-based foods, including dried fruits. Dried fruits are known to contain a diverse range of flavonoids, which contribute to their potential health benefits. Flavonoids have been extensively studied for their antioxidant and anti-inflammatory properties, as well as their ability to improve vascular health and modulate cellular signaling pathways. When consumed as part of dried fruits, flavonoids may play a role in reducing the risk of chronic diseases such as cardiovascular disease and type 2 diabetes. Additionally, flavonoids found in dried fruits have been associated with improved insulin sensitivity and glucose metabolism, which are key factors in managing diabetes. Therefore, the presence of flavonoids in dried fruits underscores their potential as a nutritious and health-promoting food choice. Several studies have reported the beneficial therapeutic effects of flavonoids in diabetes and diabetic complications [37]. A meta-analysis, comprising data from six cohort studies, demonstrated a notable link between higher total flavonoid consumption and a decreased susceptibility to T2D [38]. Excessive production of reactive oxygen species (ROS) is proposed as a significant factor contributing to the dysfunction of β-cells, which ultimately paves the path to the development of T2D. This heightened ROS generation could potentially be attributed to the activation of stress-related signaling pathways [38]. Findings derived from investigations employing cell cultures and animal models provide evidence that flavonoids possess the ability to directly neutralize ROS [39, 40]. Flavonoids exhibit the capacity to safeguard and reinstate antioxidant defense enzymes, including superoxide dismutase, catalase, and glutathione peroxidase [41]. Furthermore, they can impede the activity of enzymes that generate ROS, such as xanthine oxidase. Consequently, the presence of flavonoids results in the inhibition of various biological processes triggered by ROS, including the suppression of oxidized LDL (oxLDL)-induced cell apoptosis, as well as the modulation of NF-κB-mediated transcriptional activity, subsequently curbing inflammation [42].

Evidence drawn from multiple cohort studies has indicated that the consumption of tea, coffee, and their derivatives, characterized by their abundance in flavanols, is linked to a diminished risk of developing T2D [43–45]. Both in vitro and in vivo investigations have predominantly concentrated on grape-related research. Notably, a study led by Overman and colleagues demonstrated a substantial reduction in inflammation induced by lipopolysaccharide (LPS) through the application of grape powder extract (GPE) in macrophages. This extract also exhibited the ability to decrease the capacity of LPS-stimulated human macrophages to induce inflammation in adipocytes, subsequently alleviating the onset of insulin resistance [46]. Furthermore, the GPE exhibited an additional effect by mitigating the inflammation mediated by tumor necrosis factor-α (TNF-α) and alleviating insulin resistance (IR) in primary cultures of human adipocytes [47]. The grape polyphenol extract brought about alterations in the composition of membrane phospholipid fatty acids in an in vitro setting. Additionally, in rats subjected to a high-fat and high-sucrose diet, this extract demonstrated the ability to reduce muscle triglyceride (TG) content while concurrently increasing the expression of muscle glucose transporter type 4 (GLUT4). As a cumulative result, it led to an enhancement in IR status, as evidenced by improvements in the Homeostatic Model Assessment for Insulin Resistance (HOMA-IR) parameter [48]. This holds significant significance as the accumulation of muscle TG content and the alteration of the muscle phospholipid fatty acid profile could potentially exert an influence on lipid metabolism. This alteration in lipid metabolism could, in turn, elevate the risk of developing T2D [49]. Mice that were administered grape skin extract exhibited effects of lowering blood glucose levels (hypoglycemic) and countering excessive blood glucose levels (anti-hyperglycemic). These effects were observed to occur independently of an elevation in insulin release. Instead, it is likely that these effects are contingent upon an enhancement in insulin sensitivity, which is attributed to the activation of the insulin-signaling cascade within skeletal muscle [50]. Moreover, the grape seed aqueous extract exhibited a safeguarding effect on the pancreas against oxidative stress, inflammation, and damage caused by apoptosis. These protective actions were evident in diabetic rats, and they contributed to maintaining pancreatic function at levels close to normal [51]. (iv) Numerous dried fruits boast a wealth of antioxidant vitamins, including vitamin A, vitamin C, and vitamin E. These vitamins play a role in diminishing the risk of T2D by engaging with free radicals and thwarting oxidative harm to β-cells [52]. (v) In recent years, a growing body of research has underscored the pivotal role of the gut microbiome in shaping the development of insulin resistance and T2D. A multitude of mechanisms linked to the configuration of gut microorganisms, encompassing alterations in intestinal permeability, endotoxemia, and interactions with bile acids, have emerged as potential contributors to the initiation of insulin resistance. Additionally, the impact of dietary patterns, both over the long-term and in the short-term, on the composition of gut microbiota is firmly established [53]. Notably, specific foods may harbor a spectrum of potential prebiotic constituents. The dietary fiber present in dried fruit serves as a regulator of gut microbiota, thereby safeguarding gut health. This contribution is instrumental in lowering the risk of T2D, as it exerts an influence on various physiological processes within the host. These processes encompass lipid and glucose metabolism, as well as the maintenance of immune homeostasis [27, 54].

In summary, the therapeutic potential of dried fruits in mitigating the risk of T2D warrants further exploration. Our study adds valuable insights to this burgeoning field and underscores the importance of dietary interventions in T2D prevention and management.

### Limitation

However, the present study has some limitations: (i) This study included individuals of essentially European ancestry, so extrapolating the findings to other populations is limiting. (ii) The specific underlying mechanisms of dried fruit effects are not fully understood. (iii) The data for dried fruit intake were derived from the UK Biobank questionnaire, and therefore might be influenced by potential misclassification bias. Nevertheless, due to the large sample size, the bias would be mitigated to some extent. (iv) It is not easy to demonstrate that the results are entirely independent of the horizontal pleiotropy effect; nevertheless, we performed many sensitivity analyses to demonstrate the stability of the results.

## Conclusion

Our study identified that dried fruit intake reduces the risk of T2D through MR analysis. The results confirmed the potential benefits of dried fruit and provided some insights into daily primary prevention measures for T2D.

### Electronic supplementary material

Below is the link to the electronic supplementary material.

Supplementary Material 1

Supplementary Material 2

Supplementary Material 3

Supplementary Material 4

## Data Availability

No datasets were generated or analysed during the current study.
