# Supplementary material for: Dried fruit intake and lower risk of type 2 diabetes: a two-sample mendelian randomization study
Source: Nutr Metab (Lond). 2024 Jul 10;21:46. doi: 10.1186/s12986-024-00813-z (PMC11234600; doi:10.1186/s12986-024-00813-z)
Supplement: Supplementary file 4 — Supplementary Material 4 [file 12986_2024_813_MOESM4_ESM.docx]

***STROBE-MR***

**Supplementary Data**

Following Guides by STROBE-MR: Guidelines for strengthening the reporting of Mendelian randomization studies

1. TITLE and ABSTRACT

Manuscript title

Dried Fruit Intake and Lower Risk of Type 2 Diabetes: A two-sample Mendelian Randomization Study

Abstract

Aims:

Previous studies have shown controversy about whether dried fruit intake is associated with type 2 diabetes. This study aimed to examine the potential causal effect of dried fruit intake on type 2 diabetes by conducting a two-sample Mendelian randomization study.

Methods:

We used genome-wide association study (GWAS) summary statistics for MR analysis to explore the causal association of dried fruit intake with T2D. The inverse-variance weighted (IVW) method was used as the main analytical method for MR analysis. In addition, the MR-Egger method and the weighted median method were applied to supplement the IVW method. Furthermore, Cochrane’s Q test, MR-Egger intercept test, and leave-one-out analysis were used to perform sensitivity analysis. The funnel plot was used to assess publication bias.

Results:

The results from the IVW analysis indicated that dried fruit intake could reduce the risk of T2D [odds ratio (OR) = 0.392, 95% confidence interval (CI): 0.241–0.636, p-value = 0.0001]. In addition, the result of additional method Weighted median is parallel to the effects estimated by IVW. Furthermore, the sensitivity analysis illustrates that our MR analysis was unaffected by heterogeneity and horizontal pleiotropy. Finally, the results of the leave-one-out method showed the robustness of our MR results. And the funnel plot shows a symmetrical distribution.

Conclusions:

Our study provides evidence for the benefits of dried fruit intake on T2D. Therefore, a reasonable consumption of dried fruit may provide primary prevention.

2. Background

The consumption of dried fruits has been a topic of debate in relation to its potential impact on type 2 diabetes (T2D)[1]. Dried fruits, known for their concentrated flavors and extended shelf life, have been a popular choice among individuals seeking healthier snack alternatives. However, their high natural sugar content has raised concerns, especially in the context of T2D, a metabolic disorder characterized by impaired insulin function and elevated blood sugar level. T2D diabetes, which accounts for the majority of diabetes cases globally, poses significant challenges to public health due to its association with various complications such as cardiovascular disease, kidney dysfunction, and nerve damage[2]. As dietary choices play a crucial role in managing blood sugar levels[3], the inclusion of dried fruits in the diets of individuals with T2D diabetes has been met with both enthusiasm and caution. On one hand, dried fruits offer essential nutrients such as vitamins, minerals, and dietary fiber, making them a potentially valuable addition to a balanced diet. The fiber content in dried fruits could contribute to better blood sugar control and improved digestive health[4]. However, the concentrated sugars present in dried fruits, which are released more quickly into the bloodstream compared to their fresh counterparts, might lead to rapid spikes in postprandial glycemia, posing challenges for those aiming to maintain stable glucose levels[5]. This controversy surrounding dried fruits and T2D warrants further exploration and understanding. By analyzing the available research, we aim to provide a clearer perspective on whether dried fruits can be a suitable component of the diets of individuals with T2D, shedding light on both the potential benefits and risks associated with their consumption.

Observational studies focus only on the correlation between exposure and outcome rather than concluding causal associations. In addition, observable and unobservable residual confounders may lead to biased or opposite conclusions. Similar to randomized controlled trials, the MR study is a novel research method for exploring the causal association between exposure and outcome[6]. In MR studies, single nucleotide polymorphisms (SNPs) are considered instrumental variables (IVs) to estimate the causal association between exposures and the outcomes of interest[7]. SNPs conform to the principle of random assignment of genetic variants at meiosis, which avoids the effect of confounding factors and the potential impact of reverse causation since genetic variants precede the onset of disease[8]. A recent MR study suggests that lifestyle behaviors impact T2D risk and longevity[9, 10]. Through MR studies, more potential, influential exposure factors for T2D can be uncovered.

3. Objectives

Our study performed a two-sample MR design to investigate whether dried fruit intake is causally correlated with T2D and to provide scientific evidence for the primary prevention of T2D.

METHODS

4. Study design and data sources

The flow diagram for the whole study is shown in Figure 1. MR studies are required to satisfy the following three assumptions: (1) IVs are strongly associated with exposure factors, (2) IVs are independent of confounding factors, and (3) IVs are solely associated with outcomes through exposure factors. In our study, dried fruit intake was the exposure factor, and the outcome was T2D. In addition, fasting glucose, fasting insulin and body mass index (BMI) were considered as confounding factors.


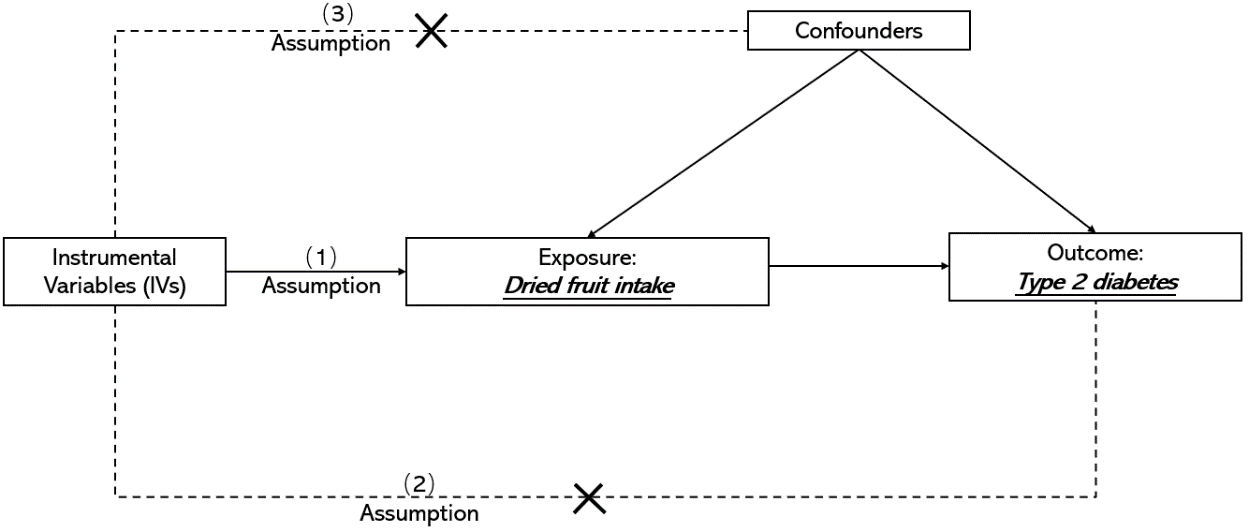


Firgure1. Flow diagram for Mendelian randomization (MR) study.

The Genome-Wide Association Study (GWAS) data for dried fruit intake were derived from a large cohort study involving approximately 500,000 individuals conducted by the UK Biobank.1 The study collected genotypic and various phenotypic data and was approved by the research ethics committee. Participants in the cohort were invited to the local evaluation center for data collection using a touch-screen questionnaire or standardized anthropometry. Participants’ intake of dried fruits as an exposure factor was extracted by a questionnaire asking about the frequency of dried fruit intake. The participants were asked, “How many pieces of dried fruit would you eat per day?” (One prune, one dried apricot and ten raisins are considered as one piece). In addition, three additional options, (1) less than one, (2) do not know, and (3) prefer not to answer, were available for participants to select. In total, 421,764 European participants’ dried fruit intake data were obtained. The GWAS summary statistics have been included in the IEU OpenGWAS database and are easily available for researchers to download (accession number: ukb-b-16576)^[11]^. Genome-wide association study summary statistics for T2D were derived from the IEU GWAS of 61,714 cases and 593,952 controls^[12]^.

5. Assumptions

MR studies are required to satisfy the following three assumptions: (1) IVs are strongly associated with exposure factors, (2) IVs are independent of confounding factors, and (3) IVs are solely associated with outcomes through exposure factors. In our study, dried fruit intake was the exposure factor, and the outcome was T2D. In addition, fasting glucose, fasting insulin and body mass index (BMI) were considered as confounding factors.

6. Statistical methods

The inverse-variance weighted (IVW) method was used as the main analytical method for estimating potential causal effects, which is an extension of the Wald ratio estimator based on the principles of Meta-analysis[14]. In addition, the MR-Egger method and the weighted median method were applied to supplement the IVW method[15, 16]. These three approaches are considered the most scientific and commonly used methods, providing robust analysis for MR studies. The criterion for using the weighted median method is that at least 50% of the SNPs must satisfy the premise that they are valid IVs[16]. The MR-Egger method provides unbiased estimates even when all selected IVs are multivariate[15]. Results of causal associations were presented as odds ratios (OR) and 95% confidence intervals (95% CI). Cochrane’s Q values were used to assess heterogeneity. MR-Egger intercept test was utilized to detect horizontal pleiotropy[17, 18]. In addition, the leave-one-out analysis was performed to assess the robustness of the results.MR estimation

7. Assessment of assumptions

We used the inverse-variance weighted (IVW) method as the primary MR approach^[13]^. MR-Egger, weighted median, and weighted mode further conducted to MR analysis. However, the estimation accuracy produced by MR-Egger is very low. Weighted median gives an accurate estimate based on the assumption that at least 50% of IVs are valid^[14]^. Weighted mode is sensitive to the difficult bandwidth selection for mode estimation^[15]^.

8. Sensitivity analyses

Cochrane’s Q values were used to assess heterogeneity. MR-Egger intercept test was utilized to detect horizontal pleiotropy[17, 18]. In addition, the leave-one-out analysis was performed to assess the robustness of the results.MR estimation

9. Software and pre-registration

All statistical analyses were conducted using the “Two Sample MR” (version 0.5.7, Stephen Burgess, Chicago, IL, USA) and “Mendelian Randomization” (version 0.8.0) in the statistical program R (version 4.3.1). p < 0.05 was considered as statistically significant. The study protocol and details were not pre-registered.

RESULTS

10. Descriptive data

We first obtained 43 SNPs that were independent of each other and strongly associated with dried fruit intake. After excluding SNPs associated with the confounding factors, 36 SNPs were finally included as IVs. Details of the 36 IVs are shown in Supplementary Table 2. The F-statistic for the 36 IVs was 15.39; thus, it can be assumed that they have a solid potential to predict the dried fruit intake level. In addition, the association of all IVs with dried fruit intake exposure was more significant than the association with T2D outcomes (Supplementary Table 3).

11. Main results

Causal effects of dried fruit intake on T2D

Mendelian randomization results from the IVW method suggest a causal association of dried fruit intake with T2D. The higher the intake of dried fruits, the lower the risk of T2D. The risk of T2D decreased by 60.8% (OR = 0.392, 95% CI: 0.241–0.636, p-value = 0.0001, Figure 2) for every increase of dried fruit intake by one standard deviation (1.275 pieces a day). Subsequently, two additional methods, MR Egger and Weighted median, were used to assess the causal association of dried fruit intake with T2D, and the result of Weighted median is parallel to the effects estimated by IVW (OR < 1) (Table 1 and Figure 3).

Table 1 Causal effects of dried fruit intake on T2D evaluated by IVW method, MR Egger method, and weighted median method.

| Outcomes | Methods | Beta | SE | OR (95% CI) | P-value |
| --- | --- | --- | --- | --- | --- |
| T2D | IVW | -0.936 | 0.247 | 0.392 (0.241,0.636) | 0.0001 |
|  | MR Egger | -0.732 | 1.179 | 0.481 (0.048,4.852) | 0.538 |
|  | Weighted median | -0.758 | 0.217 | 0.468 (0.306,0.717) | 0.0003 |

T2D, type 2 diabetes; SE, standard error; IVW, inverse variance weighted.


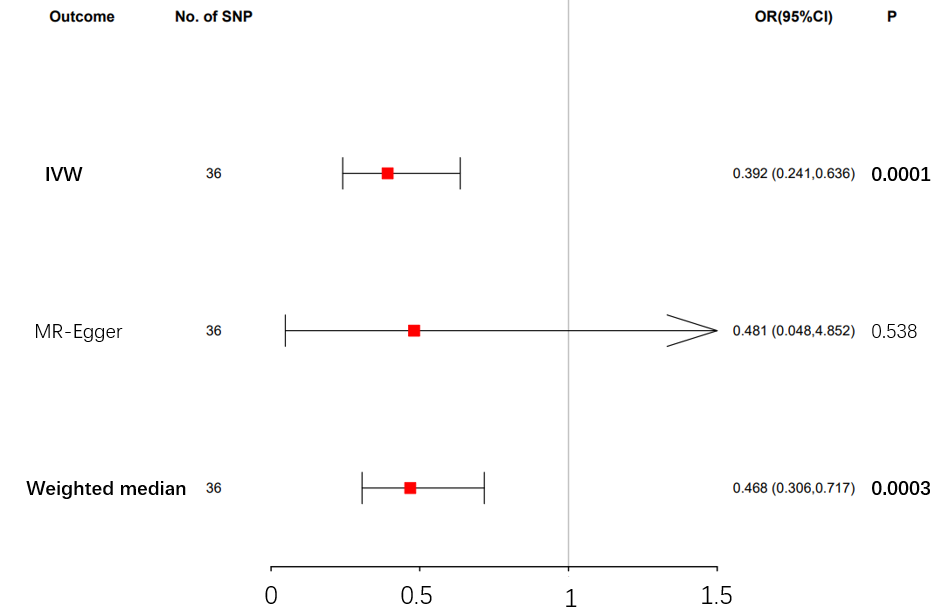


Figure 2. Causal effects of dried fruit intake on T2D assessed by the inverse-variance weighted (IVW) method, MR-Egger method, and Weighted median method.


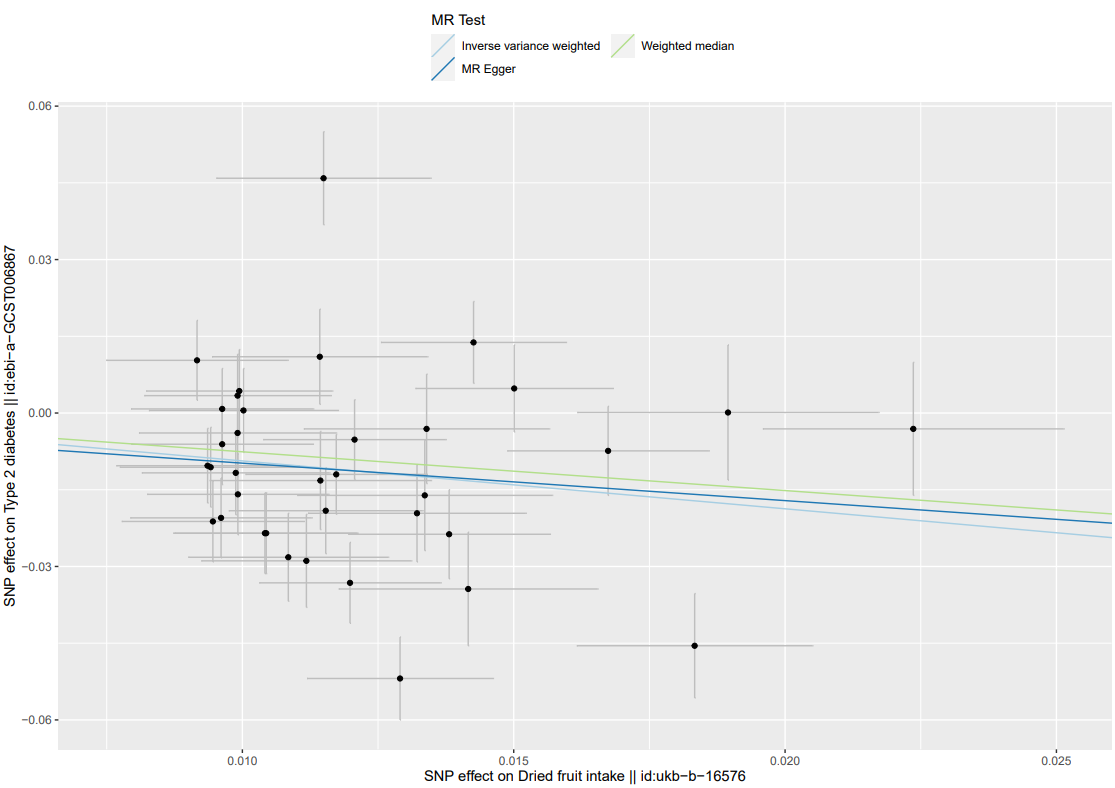


Figure 3. Scatter plot of genetic correlations of dried fruit intake and T2D using different MR methods.

13. Sensitivity and additional analyses

Cochran’s Q-test indicates significant heterogeneity among the 36 IVs. (Q_pvalIVW = 7.840342e-16, Q_pvalMR−Egger = 3.904277e-16) (Table 2). In addition, the results of the MR-Egger intercept test suggested that there was no horizontal pleiotropy between dried fruit intake and T2D. (MR-Egger intercept = -0.0025, p-value = 0.86) Moreover, the leaveone-out analysis demonstrated the stability of the MR results in our study since excluding any one IV did not shift the overall results (Figure 4). And the funnel plot shows a symmetrical distribution. (Figure 5)

Table 2 Results of heterogeneity by Cochran’s Q test.

| Outcome | Method | Cochran’s Q test | | |
| --- | --- | --- | --- | --- |
|  |  | Q | Q_df | Q_pval |
| T2D | IVW | 147.91 | 34 | 7.84e-^16^ |
|  | MR-Egger | 147.77 | 35 | 3.904e-^16^ |

T2D, type 2 diabetes; IVW, inverse variance weighted.

DISCUSSION

14. Key results

In the light of past medical opinion, the consumption of dried fruit was discouraged because of their high fat and sugar content. However, this view has been overturned in recent years. Conventional dried fruits exhibit a notable fiber content, minimal fat presence, and serve as a concentrated reservoir of diverse micronutrients. Moreover, they offer enhanced convenience and extended shelf life when juxtaposed with their fresh fruit counterparts[19]. For instance, numerous randomized clinical trials and animal studies have underscored the potential advantageous impact of dried fruits in mitigating cardiovascular diseases[20, 21]. Dried fruits encompass a spectrum of macro and micronutrients, accompanied by significant bioactive compounds, which possess the potential to collaboratively influence and modulate distinct metabolic ailments[21-23]. Nonetheless, the linkage between dried fruit consumption and T2D has been scarcely explored in existing research. Consequently, the precise involvement of dried fruit in the onset and advancement of T2D remains a subject of dispute[25] and the potential of dried fruit intake to mitigate T2D risk lacks consensus. To address this gap, our study represents a pioneering effort in the field of MR, being the inaugural MR investigation aimed at evaluating the causal impact of dried fruit consumption on the development of T2D.

Our study strictly followed the three main assumptions of the MR study. For assumption 1, we adopted a strict threshold of p-value < 5 × 10−8 to screen SNPs associated with dried fruit intake as IVs. In addition, we eliminated the linkage disequilibrium of IVs. Furthermore, the F-statistic of IVs is greater than 10. For assumption 2, we downloaded GWAS summary statistics of confounders (common T2D risk factors) and excluded SNPs from the IVs that were strongly associated with these confounders (p-value < 5 × 10−8). Finally, for assumption 3, all IVs were more strongly correlated with dried fruit exposure factors than with T2D outcomes. In addition, the MR-Egger intercept test suggested that the results were not influenced by horizontal pleiotropy (p-value > 0.05). And the present study demonstrates that dried fruits intake may reduce the risk of T2D.

15. Limitations

However, the present study has some limitations: (i) This study included individuals of essentially European ancestry, so extrapolating the findings to other populations is limiting. (ii) The specific underlying mechanisms of dried fruit effects are not fully understood. (iii) The data for dried fruit intake were derived from the UK Biobank questionnaire, and therefore might be influenced by potential misclassification bias. Nevertheless, due to the large sample size, the bias would be mitigated to some extent. (iv) It is not easy to demonstrate that the results are entirely independent of the horizontal pleiotropy effect; nevertheless, we performed many sensitivity analyses to demonstrate the stability of the results.

16. Interpretation

We speculate several potential mechanisms may explain this finding. (i) A secondary analysis of data extracted from the National Health and Nutrition Examination Survey provided evidence indicating a correlation between the consumption of dried fruits and positive outcomes, including enhanced nutrient intake, elevated composite diet quality scores, and reduced BMI. It is noteworthy that obesity, recognized as a prevalent risk factor for T2D, is implicated in this context^[26-28]^. Previous studies have revealed that a poor diet quality score leads to an increased risk of T2D^[29]^. (ii) Hyperglycemia exerts detrimental impacts on the functional capacity of pancreatic β-cells, resulting in their dysfunction and concurrent development of insulin resistance, ultimately culminating in the onset of diabetes mellitus. In support of this, an animal study revealed the potential protective attributes of hawthorn fruit extract. This extract demonstrated the ability to mitigate high fructose-induced oxidative stress and endoplasmic reticulum stress specifically within pancreatic β-cells^[30]^. (iii) Dried fruit is a matrix of important bioactive compounds such as Vitamins (Vitamin E, niacin, choline and/or folic acid)^[31]^, micronutrient minerals (magnesium, potassium, calcium and/or phosphorus)^[32]^, phenolic compounds, and carotenoid^[33]^. Certain constituents within dried fruits have the potential to exert a pivotal influence on mitigating the risk of T2D. For example, carotenoids present in dried fruits possess inherent antioxidant attributes that may contribute to diminishing the susceptibility to diabetes. Notably, augmented dietary intake of carotenoids is correlated with a lowered risk of developing T2D, underscoring the significant relationship between higher carotenoid consumption and risk reduction^[34]^. Dried fruits stand out due to their substantial β-carotene content, a factor that has been extensively linked to a protective role against the development of T2D^[27, 35]^. Furthermore, these fruits predominantly consist of carbohydrates, with relatively low proportions of protein and fat. Importantly, both dried fruits and their counterparts comprise a notable quantity of dietary fiber. This distinctive and diverse nutritional composition positions dried fruits as essential dietary components to mitigate the risk of various metabolic disorders^[36]^. Several studies have reported the beneficial therapeutic effects of flavonoids in diabetes and diabetic complications^[37]^. Additionally, a meta-analysis, comprising data from six cohort studies, demonstrated a notable link between higher total flavonoid consumption and a decreased susceptibility to T2D^[38]^. Excessive production of reactive oxygen species (ROS) is proposed as a significant factor contributing to the dysfunction of β-cells, which ultimately paves the path to the development of T2D. This heightened ROS generation could potentially be attributed to the activation of stress-related signaling pathways^[38]^. Findings derived from investigations employing cell cultures and animal models provide evidence that flavonoids possess the ability to directly neutralize ROS^[40]^. Flavonoids exhibit the capacity to safeguard and reinstate antioxidant defense enzymes, including superoxide dismutase, catalase, and glutathione peroxidase^[41]^. Furthermore, they can impede the activity of enzymes that generate ROS, such as xanthine oxidase. Consequently, the presence of flavonoids results in the inhibition of various biological processes triggered by ROS, including the suppression of oxidized LDL (oxLDL)-induced cell apoptosis, as well as the modulation of NF-κB-mediated transcriptional activity, subsequently curbing inflammation^[42]^.

Evidence drawn from multiple cohort studies has indicated that the consumption of tea, coffee, and their derivatives, characterized by their abundance in flavanols, is linked to a diminished risk of developing T2D^[43-45]^. Both in vitro and in vivo investigations have predominantly concentrated on grape-related research. Notably, a study led by Overman and colleagues demonstrated a substantial reduction in inflammation induced by lipopolysaccharide (LPS) through the application of grape powder extract (GPE) in macrophages. This extract also exhibited the ability to decrease the capacity of LPS-stimulated human macrophages to induce inflammation in adipocytes, subsequently alleviating the onset of insulin resistance^[46]^. Furthermore, the GPE exhibited an additional effect by mitigating the inflammation mediated by tumor necrosis factor-α (TNF-α) and alleviating insulin resistance (IR) in primary cultures of human adipocytes^[47]^. The grape polyphenol extract brought about alterations in the composition of membrane phospholipid fatty acids in an in vitro setting. Additionally, in rats subjected to a high-fat and high-sucrose diet, this extract demonstrated the ability to reduce muscle triglyceride (TG) content while concurrently increasing the expression of muscle glucose transporter type 4 (GLUT4). As a cumulative result, it led to an enhancement in IR status, as evidenced by improvements in the Homeostatic Model Assessment for Insulin Resistance (HOMA-IR) parameter^[48]^. This holds significant significance as the accumulation of muscle TG content and the alteration of the muscle phospholipid fatty acid profile could potentially exert an influence on lipid metabolism. This alteration in lipid metabolism could, in turn, elevate the risk of developing T2D^[49]^. Mice that were administered grape skin extract exhibited effects of lowering blood glucose levels (hypoglycemic) and countering excessive blood glucose levels (anti-hyperglycemic). These effects were observed to occur independently of an elevation in insulin release. Instead, it is likely that these effects are contingent upon an enhancement in insulin sensitivity, which is attributed to the activation of the insulin-signaling cascade within skeletal muscle^[50]^. Moreover, the grape seed aqueous extract exhibited a safeguarding effect on the pancreas against oxidative stress, inflammation, and damage caused by apoptosis. These protective actions were evident in diabetic rats, and they contributed to maintaining pancreatic function at levels close to normal^[51]^. (iv) Numerous dried fruits boast a wealth of antioxidant vitamins, including vitamin A, vitamin C, and vitamin E. These vitamins play a role in diminishing the risk of T2D by engaging with free radicals and thwarting oxidative harm to β-cells^[52]^. (v) In recent years, a growing body of research has underscored the pivotal role of the gut microbiome in shaping the development of insulin resistance and T2D. A multitude of mechanisms linked to the configuration of gut microorganisms, encompassing alterations in intestinal permeability, endotoxemia, and interactions with bile acids, have emerged as potential contributors to the initiation of insulin resistance. Additionally, the impact of dietary patterns, both over the long-term and in the short-term, on the composition of gut microbiota is firmly established^[53]^. Notably, specific foods may harbor a spectrum of potential prebiotic constituents. The dietary fiber present in dried fruit serves as a regulator of gut microbiota, thereby safeguarding gut health. This contribution is instrumental in lowering the risk of T2D, as it exerts an influence on various physiological processes within the host. These processes encompass lipid and glucose metabolism, as well as the maintenance of immune homeostasis^[27, 54]^.

OTHER INFORMATION

17.Data availability statement

Publicly available datasets were analyzed in this study. This data can be found here: IEU Open GWAS project (https://gwas.mrcieu.ac.uk/).

18.Ethics statement

The studies involving human participants were reviewed and approved by Local Ethics Committees of consortia in the respective studies. The patients/participants provided their written informed consent to participate in this study.

19.Author contributions

JBG designed the study, analyzed the data, and wrote the manuscript. TL, JBG and HC assisted in analyzing the data and revising the manuscript. KTY critically read and edited the manuscript. All authors contributed to the article and approved the submitted version.

20.Funding Not applicable.

21.Acknowledgments

We want to acknowledge the participants and investigators of UK Biobank. We are grateful to the IEU Open GWAS Project for providing the summary GWAS statistics from UK Biobank.

22.Conflict of interest

The authors declare that the research was conducted in the absence of any commercial or financial relationships that could be construed as a potential conflict of interest.
